# The effects of upper and lower limb elastic band training on the change of direction, jump, power, strength and repeated sprint ability performance in adolescent female handball players

**DOI:** 10.3389/fspor.2023.1021757

**Published:** 2023-02-22

**Authors:** Nawel Gaamouri, Mehrez Hammami, Yosser Cherni, Dustin J. Oranchuk, Nicola Bragazzi, Beat Knechtle, Mohamed Souhaiel Chelly, Roland van den Tillaar

**Affiliations:** ^1^ Research Unit (UR17JS01) “ Sport Performance, Health & Society”, Higher Institute of Sport and Physical Education of Ksar Saîd, University of “La Manouba”, Tunis, Tunisia; ^2^ Higher Institute of Sport and Physical Education of Ksar Said, University of “La Manouba”, Tunis, Tunisia; ^3^ Sports Performance Research Institute New Zealand, Auckland University of Technology, Auckland, New Zealand; ^4^ Department of Mathematics and Statistics York University Toronto, Laboratory for Industrial and Applied Mathematics (LIAM), York University, Toronto, ON, Canada; ^5^ Institute of Primary Care, University of Zurich, Zurich, Switzerland; ^6^ Medbase St. Gallen Am Vadianplatz, St. Gallen, Switzerland; ^7^ Department of Sports Sciences and Physical Education, Nord University, Levanger, Norway

**Keywords:** 1-RM half squat, force-velocity test, team sports, resistance training, standing long jump

## Abstract

This study aimed to examine the effects of incorporating 10-week elastic band strength training (EBST) program on change of direction, jumping ability, repeated sprint ability, and both muscular strength and power in adolescent female handball players. Participants aged 15.8 ± 0.2 years were divided by playing position, and players from each position were then randomly assigned between the elastic strength (*n* = 17) and control (*n* = 17) groups. The experimental group performed periodized upper and lower-body elastic band strength training twice weekly for ten weeks by substitution of some of their regular physical and game preparation. The control group only performed regular handball training. Two-way analyses of variance (group × time) assessed change of direction (COD) *via* the modified *t*-agility test, squat jump, countermovement jump, standing long jump, repeated sprint ability (RSA), 1-RM bench press and half squat, and cycle ergometer force-velocity tests for both upper and lower limbs. Relative to the control group, the experimental group enhanced COD performance [*p* < 0.001; Cohen's effect size (d) = 1.00]; squat and countermovement jump (*p* = 0.002, d ≥ 0.83), best, mean, and total RSA scores (all *p* < 0.001, d = 0.92–1.66), 1-RM bench press (*p* = 0.02, d = 0.59) and half squat (*p* = 0.009, d = 0.67), all indices of upper limb force-velocity performance (*p* ≤ 0.025, d = 0.56–1.66), and 3 of 4 indices of lower limb force-velocity performance (*p* ≤ 0.004, d = 0.75–0.92). We conclude that additional elastic band training performed twice a week for ten weeks improves measures relevant to handball game performance in adolescent female athletes.

## Introduction

Handball is a team sport characterized by repeated high-threshold actions such as sprinting, jumping, change of direction, throwing, blocking, making physical contacts, passing the ball, and attempting to establish an optimal position for the throwing player (1–3). The development of these particular skills and the associated high levels of strength and power is a focus for coaches and sports scientists (4–11). Indeed, Ratel (12) suggested that muscle strength and power are key components of fitness performance and are required in many of the aforementioned actions (e.g., sprinting, jumping, change of direction); constituting an essential part of any young athlete's overall training program (4–8, 10),.

A better understanding of the physical capacities underlying handball could greatly facilitate the planning of training interventions to optimize handball players' physical performance and preparation. Strength training with different forms (i.e., resistance training with external load, plyometric training, complex training, contrast training) is the most commonly used method of improving physical performance in handball (4–8, 10, 11, 13–15). However, there is also interest in simple, inexpensive, portable and effective conditioning techniques such as elastic bands. Elastic band strength training is a good alternative to traditional strength training equipment (4, 6, 10, 16–18). Mascarin, de Lira (10) noted a significant increase in handball throwing velocities (standing and jump shot) and isokinetic shoulder strength after six weeks of tri-weekly resistance band training for the shoulders in young female handball players. Similarly, Andersen, Fimland (6) revealed that elastic band training, incorporated into regular handball training sessions, improved explosive lower-limb performance in young female handball players than handball training alone. Given the potential of elastic band training in the development of physical capabilities in handball, there seems to be a lack in the literature investigating this type of training in youth female handball athletes. Furthermore, no study examined the effect of elastic band training in repeated sprint ability in young female athletes.

Few previous studies have examined the impact of elastic band training on young female handball players (6, 10), and this is the first report to have examined the effect of upper and lower elastic band training upon power using force velocity test. Therefore, this study aimed to determine how far the substitution of a short-term elastic band strength training program for some existing drills within a regular in-season handball training program would enhance the high-intensity actions of adolescent female handball players. A combined lower-limb and upper-limb elastic band training program were introduced into the regular in-season regimen for ten weeks for experimental subjects without increasing their total training time. We hypothesized that the EBST intervention would positively augment both upper and lower limbs performance, increasing jump, change-of-direction, muscular strength, muscular power and repeated sprint performance relative to players who performed standard in-season training.

## Materials and methods

### Participants

Thirty-four female adolescent handball players from two teams (i.e., both teams played in the first national league and followed nearly identical training and competition schedule) participated in this study, The selected team members were matched according to field positions and randomly distributed between the elastic strength group (experimental group; *n* = 17) and control group (*n* = 17) (basal physical characteristics in Table 1). Written informed consent was obtained from all subjects' parents or guardians (and consent from the athletes) before participating in the study, approved by the Local Ethics Committee Research Unit (UR17JS01) in conformity with principles identified in the latest version of the Declaration of Helsinki.

**Table 1:** Physical characteristics of experimental and control groups (mean ± SD).

	Elastic band training (*n* = 17)	Control (*n* = 17)
Age (years)	15.7 ± 0.2	15.8 ± 0.2
Body mass (kg)	63.4 ± 3.8	63.0 ± 3.8
Height (m)	1.69 ± 0.42	1.67 ± 0.35
Body fat (%)	21.5 ± 1.8	21.7 ± 2.5
Age of peak height velocity (years)	12.4 ± 0.4	12.8 ± 0.3
Predicted years from age of peak height velocity	3.3 ± 0.5	3.0 ± 0.4

**Figure 1:**
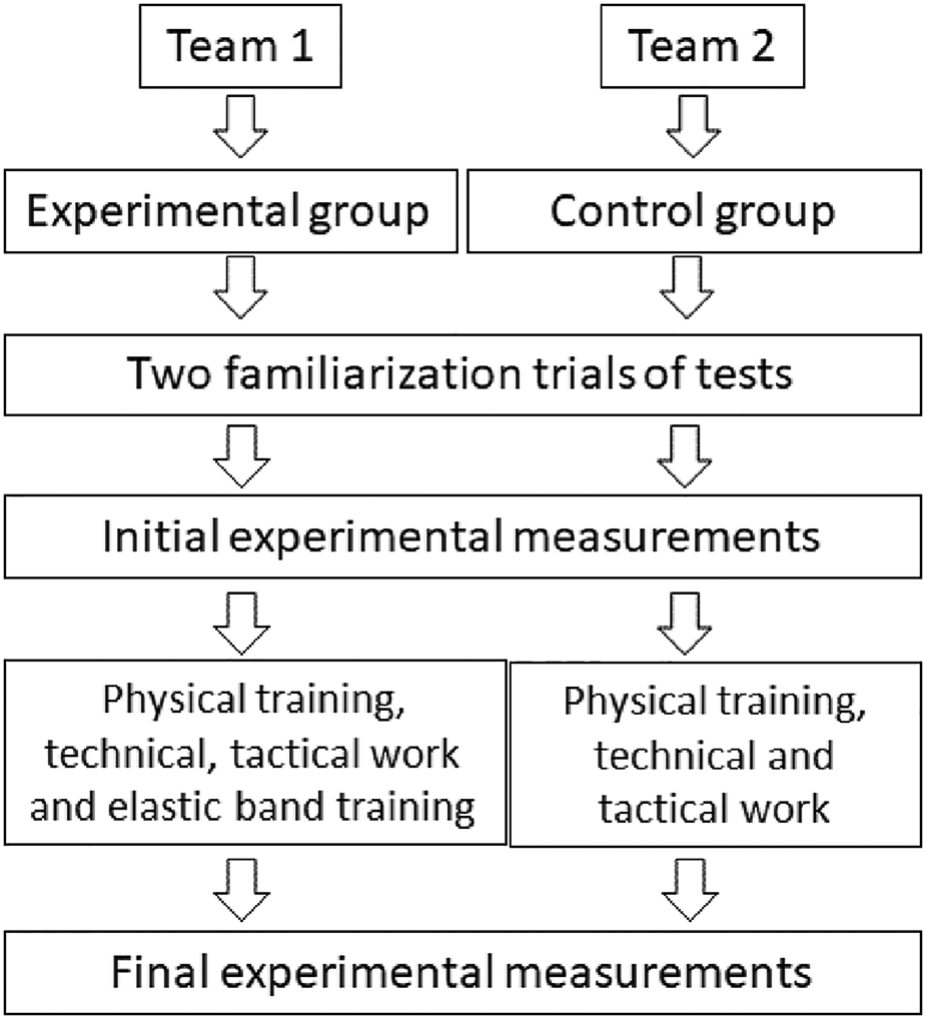
The diagram includes detailed information on the interventions received.

All participants had a competitive experience of ≥5 years and had reached a medium competitive level within their division (i.e., U-17 National League). During the four months preceding the intervention, they trained six times per week (∼2 h per session) and competed once per week. The 10-wk intervention was carried out during the regular season (i.e., in-season). Goalkeepers were excluded from the study because they did not participate in the same physical training program as the other players. During the intervention, both the control and experimental group athletes continued their regular fitness sessions. However, the experimental group replaced a part of their technical/tactical sessions with elastic band training (Figure 1).

### Testing procedures

All measures were performed 1-wk before and four days after the last elastic band training session. The variables examined included change of direction (COD) [(Modified agility *T*-test], three jumping tests [squat jump, countermovement jump and standing long jump], repeated sprint ability, muscular strength [1-RM bench press and 1-RM half squat] and muscular power [force-velocity test for both upper and lower limb]. Two familiarization sessions of 60–70 min preceded testing. Data were collected at the same time of day, under similar environmental conditions, and separated at least 48 h from familiarization sessions. During the 24 h before testing sessions, players avoided strenuous training and forms were delivered to all players by a certificate nutritionist with instructions regarding what to eat to follow a carbohydrate-rich diet. No caffeine-containing products were consumed for three h before testing. A standardized warm-up (10–20 min of low- to moderate-intensity aerobic exercise and dynamic stretching) preceded all the tests.

### Anthropometry

Anthropometric measurements included standing and sitting body height (stadiometer accuracy of 0.1 cm; Holtain, Crosswell, Crymych, Pembs, United Kingdom) and body mass (0.1 kg; Tanita BF683W scales, Munich, Germany). The overall percentage of body fat was estimated from the biceps, triceps, subscapular, and suprailiac skinfolds, using the equations Durnin and Womersley (19) for children and adolescent females:

%Bodyfat=(495/D)−450

where D = 1.1369–0.0598 (Log sum of 4 skinfolds)

Maturity offset status was calculated from peak height velocity (20):

Maturity offset = −9.38 + (0.000188 × leg length × sitting height) + (0.0022 × age × leg length) + (0.00584 × age × sitting height) + (0.0769 × weight/height ratio).

### Modified change of direction *T*-test (T-half)

The T-half was performed as previously described (21) to determine the speed with directional changes such as forward sprinting, left and right shuffling, and backpedalling. Performance times were recorded to the nearest 0.01 s by paired single beam photocells (Microgate, Bolzano, Italy). Each player performed two attempts with 5 min between them, and the best attempt was used for analyses.

### Vertical jumps

Before the test, a weighted resistance warm up constituted by 5 countermovement jumps onto a box, with the athletes holding dumbbells equaling 10% of their body weight exercises and 5 countermovement jumps at 75% intensity of their past maximum vertical jump score exercises (22). Jump height was assessed using an infrared photocell mat connected to a digital computer (Optojump System, Microgate SARL, Bolozano, Italy). Flight times were measured with a precision of 1/1,000 s, allowing calculation of jump heights. Subjects began the squat jump at a knee angle of 90° and performed a vertical jump by pushing upwards, keeping their legs straight. The countermovement jump (CMJ) began from an upright position, with subjects making a rapid downward movement to a knee angle of 90° (again self-monitored, using a mirror and with the experimenter) and rapidly reversing directions to jump vertically maximally. Each player performed three attempts with 5 min between them, and the average of three attempts was used for analyses.

### Standing long jump

The standing long jump start position required subjects to stand with their feet at shoulders' width behind a line marked on the ground and their arms in a neutral position. On the command “ready, set, go”, participants executed a countermovement with their legs and arms and jumped at maximal effort in the horizontal direction. Participants had to land with both feet simultaneously and were not allowed to fall forward or backward. The horizontal distance between the starting line and the heel of the rear foot was recorded *via* tape measure to the nearest 1 cm.

### Repeated sprint ability

After a standardized warm-up, the shuttle repeated sprint ability test involved six repetitions of 2 × 20 m shuttle sprints (approximately 7 s running time). For both tests, sprints were repeated every 20 s (23). A brisk walk back to the starting line allowed active recovery. Three seconds before starting each sprint, subjects took an individually chosen starting position 0.5 m behind the timing gate. A digital timer started automatically when the player passed the gate. Two timing-gates (Microgate Srl; Race time 2. Light Radio, Bolzano, Italy) working in opposite directions allowed subjects to start the next run from the end where they had finished the preceding sprint. Strong verbal encouragement was provided throughout, and subjects were instructed to perform each sprint with maximal effort. Four scores were calculated: best sprint time, mean sprint time, total sprint time and fatigue index, the last calculated as the percentage decrement: 100–(total time/ideal time × 100); where the ideal time = 6 × best time during test (23).

### 1-RM Half squat and bench press

1-RM was used to assess the participants’ muscular strength (24) and was measured before the training program's beginning and re-assessed at weeks 4 and 10. Thus, training loads (%RM) were accurately adjusted during the training program, following previous literature guidelines (25). First, the player was instructed to perform a light resistance warm-up from 10 to 12 repetitions in the assessed exercise, followed by 1-minute of passive rest. A warm-up load was added to allow the athlete to complete 3–5 repetitions (5%–10% for bench press and 10%–20% for back squat). A 2-minute rest period was provided. An additional 5%–10% increase in the load was performed for the bench press and 10%–20% for the half squat, followed by a 4-minute rest period. The load continued to be increased until the player completed 1-RMs with proper exercise technique, as determined by an experienced strength and conditioning coach. In the back squat, participants were asked to descend until the knee joint reached a 90° angle. In the bench-press, the elbow joint was required to reach a 90° angle.

### The force–velocity test

The lower limb force–velocity tests were performed on a standard cycle ergometer (model 894 E, Monark Exercise AB, Vansbro, Sweden). The instantaneous peak velocity at each braking force was used to calculate the corresponding maximal anaerobic power. The maximal velocity (V0) was defined as the highest velocity attained without external loading. Peak power was defined as the power at which additional loading induced a decrease in power output. Parabolic relationships were calculated only if a decline of peak power over two successive braking forces was observed.

Upper limb tests were made using an appropriately modified version of the same apparatus. Hand cranks replaced the pedals, and the saddle pillar was removed to avoid injuries. The ergometer was then mounted on a metal support that brought the crankshaft to shoulder level. The unrestrained subjects stood freely in front of the ergometer, except smaller participants were allowed to stand on a step.

The measured and calculated parameters for both tests included the peak power of the upper and lower limbs, each expressed in Watts, and W·kg^−1^ of total body mass, and the corresponding maximal theoretical forces and maximal velocities. The force-velocity tests required short, all-out sprints (duration ∼7 s) using a suitable sequence of ergometer braking forces (26, 27). After a 10-minute standardized warm-up, lower limbs tests began at a braking force equal to 2.5% of the participant's body mass (26). After a 5-minute recovery, the braking was increased to 5%, 7.5%, 8.5%, 9.5%, 10.5%, and 11.5% of body mass in randomized order. The exact sequence was performed again until an additional load induced a decrease of power during two consecutive repetitions; this value was accepted as the peak power. Six to 8 all-out sprints were generally performed in a session. The upper limbs protocol was similar, beginning with a braking force equal to 1.5% of the participant's body mass. After a 10-minute warm-up, the braking was increased by 0.5% every bout until the subject could not reach the previous peak of power in 2 successive bouts.

### Elastic band strength training program

The training intervention consisted of a progressive 10-week upper and lower body elastic band training program. Training was completed during the mid-portion of the 2018–2019 competitive season (January to March). The design of the intervention was based on the players’ previous training records and research results (6, 10, 16, 28) (Table 2). Bi-weekly elastic band sessions (Tuesdays and Thursdays) included four exercises for the upper limb and four exercises for the lower limb. The elastic band system (Thera-Bands; Hygenic Corporation, Akron, OH, United States) included four latex bands of differing elasticity: red [250% elongation (3.2 kg)], green [250% elongation (4.4 kg)], blue [250% elongation (6 kg)] and black [250% elongation (8 kg)]. Upper limb exercises included flies, rows with high elbows, trunk rotation, and standing press. Lower limb exercises involved knee extension, knee flexion, half squat, and hip adduction. The exercise order alternated each session (upper limb exercise then lower limb exercise). The elastic band was folded to double its resistance to extension in the lower limb exercise but not double for the upper limb exercise. The necessary amplitudes of movement during each exercise were calculated individually, thus determining appropriate attachments of the bands to the wall and the player's body. Recovery between sets was 30 s. All exercises were performed with the maximal effort level. The initial length of the elastic band was 120 cm for all exercises. The elastic band training was not added to the regular handball training but was immediately performed after the warm-up programme (6) to replace some low-intensity technical-tactical handball drills. Elastic band training activity accounted for <10% of the total handball-training load (competitive and friendly matches not accounted for). The control group subjects followed their regular handball training (i.e., mainly technical-tactical drills, small-sided and simulated games, and injury prevention drills). The overall handball training load was comparable between groups (using the Borg Rating of Perceived Exertion). They were following similar handball training routines consisting of 6 sessions per week with 90–120 min each.

**Table 2:** Elastic band strength training program.

Exercises	Week 1	Week 2	Week 3	Week 4	Week 5	Week 6	Week 7	Week 8	Week 9	Week 10
Upper limb	Red elastic band at 250% elongation (3.2 kg)	Green elastic band at 250% elongation (4.4 kg)			Blue elastic band at 250% elongation (6 kg)			Black elastic band at 250% elongation (8 kg)		
	Sets × Reps	Sets × Reps	Sets × Reps	Sets × Reps	Sets × Reps	Sets × Reps	Sets × Reps	Sets × Reps	Sets × Reps	Sets × Reps
Flies	3 × 10	3 × 10	4 × 10	5 × 10	3 × 10	4 × 10	5 × 10	3 × 10	4 × 10	5 × 10
High elbow row	3 × 10	3 × 10	4 × 10	5 × 10	3 × 10	4 × 10	5 × 10	3 × 10	4 × 10	5 × 10
Trunk rotation	3 × 10	3 × 10	4 × 10	5 × 10	3 × 10	4 × 10	5 × 10	3 × 10	4 × 10	5 × 10
Standing press	3 × 10	3 × 10	4 × 10	5 × 10	3 × 10	4 × 10	5 × 10	3 × 10	4 × 10	5 × 10
Lower limb	Red elastic band “Folding” at 250% elongation (6.4 kg)	Green elastic band “Folding” at 250% elongation (8.8 kg)			Blue elastic band “Folding” at 250% elongation (12 kg)			Black elastic band “Folding” at 250% elongation (16 kg)		
	Sets × Reps	Sets × Reps	Sets × Reps	Sets × Reps	Sets × Reps	Sets × Reps	Sets × Reps	Sets × Reps	Sets × Reps	Sets × Reps
Knee extension	3 × 10	3 × 10	4 × 10	5 × 10	3 × 10	4 × 10	5 × 10	3 × 10	4 × 10	5 × 10
Knee flexion	3 × 10	3 × 10	4 × 10	5 × 10	3 × 10	4 × 10	5 × 10	3 × 10	4 × 10	5 × 10
Half squat	3 × 10	3 × 10	4 × 10	5 × 10	3 × 10	4 × 10	5 × 10	3 × 10	4 × 10	5 × 10
Hip adduction	3 × 10	3 × 10	4 × 10	5 × 10	3 × 10	4 × 10	5 × 10	3 × 10	4 × 10	5 × 10

N.B.: the overall handball training load was comparable between the groups (using the Borg Rating of Perceived Exertion).

“Folding”: the elastic band was folded to double its resistance to extension in the lower limb exercise.

### Statistical analyses

Statistical analyses were carried out using the SPSS 22 program for Windows (SPSS, Inc., Armonk, NY: IBM Corp). Normality of all variables was tested using the Kolmogorov–Smirnov test procedure. Data are presented as mean ± standard deviation (SD) and as median values for skewed variables. Between-group differences at baseline were examined using independent *t*-tests, and the effect of the intervention was determined by 2-way analyses of variance [Experimental vs. Control and pre vs. posttest]. Effect sizes were calculated by converting partial eta squared values to Cohen's *d* [classiﬁed as small (0.00 ≤ *d *≤ 0.49), medium (0.50 ≤ *d *≤ 0.79), and large (*d *≥ 0.80)] (29). The criterion for statistical significance was set at *p *< 0.05, whether a positive or a negative difference was seen (i.e., a 2-tailed test was adopted). The reliabilities of all dependent variables were assessed by calculating intra-class correlation coefficients (ICC) (2-way mixed) and coefficients of variation (%CV) to evaluate intra-session reliability.

## Results

No athletes missed more than 10% of the total training sessions or more than two consecutive sessions, so it was unnecessary to exclude any participants from the study. Test-retest reliabilities were generally above the accepted threshold, with ICCs of 0.93–0.98 and CVs of 2.1%–9.2% (Table 3). There were no signiﬁcant initial intergroup differences for any of the dependent variables.

**Table 3:** Reliability and variability of performance tests.

	ICC	95% confidence interval
T-half test	0.982	0.96–0.99
Standing long jump	0.989	0.98–0.99
Squat jump	0.926	0.85–0.96
Countermovement jump	0.897	0.79–0.95

Most variables for both groups increased significantly over the 10-week intervention. A significant group × time interaction was found for almost all variables. The experimental group enhanced T-half performance, vertical jump performance, all repeated sprint time scores except fatigue index, strength, all force-velocity scores for the upper limb performance and for the lower body peak power and maximal force scores more compared to the control group with medium to large effect sizes (Table 4).

**Table 4:** Means and standard deviations for all outcome measures before (Pre) and after (post) the intervention period in the experimental and control groups.

	Experimental group (*n* = 17)					Control group (*n* = 17)					group x time interaction	
	Pre	Post	% Δ change	Paired *t* test		Pre	Post	%Δ change	Paired *t* test		*p*	Cohen's *d*
				*p*	Cohen's *d*				*p*	Cohen's *d*		
**Change of direction**
*T*-test (s)	7.45 ± 0.16	7.02 ± 0.26	−5.8 ± 3.6	<0.001	2.05	7.46 ± 0.17	7.40 ± 0.18	−0.7 ± 0.8	0.001	0.35	<0.001	1.00
**Jump**
Squat jump (cm)	21.9 ± 1.7	25.5 ± 1.2	17 ± 6.5	<0.001	−2.52	22 ± 2.1	23 ± 1.6	4.7 ± 4.4	<0.001	−0.55	0.002	0.82
Counter movement jump (cm)	22.8 ± 1.8	26.8 ± 2	17.7 ± 5.1	<0.001	−2.17	22.9 ± 2	23.8 ± 1.8	4.2 ± 3.5	<0.001	−0.49	0.002	0.83
Standing long jump (cm)	146 ± 13	156 ± 12	6.9 ± 3.5	<0.001	−0.82	146 ± 15	157 ± 16	7.6 ± 7.6	0.002	−0.73	0.882	0.00
**Repeated sprint ability**
Best time (s)	7.55 ± 0.07	7.31 ± 0.07	−3.1 ± 0.4	<0.001	3.53	7.54 ± 0.06	7.50 ± 0.06	−0.5 ± 0.3	<0.001	0.69	<0.001	0.92
Average time (s)	7.66 ± 0.06	7.43 ± 0.06	−3.1 ± 0.3	<0.001	3.95	7.69 ± 0.07	7.66 ± 0.05	−0.4 ± 0.5	0.001	0.51	<0.001	1.52
Total time (s)	46.0 ± 0.4	44.6 ± 0.4	−3.1 ± 0.3	<0.001	3.61	46.2 ± 0.4	45.9 ± 0.3	−0.4 ± 0.5	0.002	0.87	<0.001	1.66
Fatigue index (%)	1.53 ± 0.43	1.58 ± 0.55	3.90 ± 24.6	0.609	−0.04	2.03 ± 0.63	2.10 ± 0.52	7.5 ± 25	0.523	−0.12	0.941	0.00
**1-Repetition maximum**
Bench press (kg)	38.4 ± 9.2	54.7 ± 11	47.4 ± 37.4	<0.001	−1.66	35.3 ± 9.6	38.7 ± 9.9	10.2 ± 5.5	<0.001	−0.36	0.009	0.67
Half squat (kg)	78.7 ± 11	95.8 ± 8.5	22.7 ± 8.8	<0.001	−1.79	73.8 ± 12.7	77.6 ± 13.2	5.8 ± 10.5	0.039	−0.30	0.020	0.59
**Upper limb**
Peak power (W)	149 ± 22	218 ± 24	49.3 ± 2.3	<0.001	−3.12	146 ± 22	157 ± 34	9.0 ± 27.6	0.269	−0.37	<0.001	1.54
Peak power per body mass (W/kg)	2.3 ± 0.3	3.4 ± 0.3	46.1 ± 26.5	<0.001	−3.78	1.9 ± 0.2	2.0 ± 0.3	5.7 ± 18.5	0.342	−0.40	<0.001	1.66
Maximal pedaling velocity (km/h)	90 ± 16	103 ± 12	15.0 ± 19.3	<0.001	−0.91	87 ± 17	84 ± 8	−0.3 ± 21	0.473	0.25	0.026	0.56
Maximal braking force (N/kg)	6.7 ± 1.0	8.7 ± 1.3	31.8 ± 21.0	<0.001	−1.78	6.7 ± 1.3	7.3 ± 0.9	11.7 ± 22.4	0.049	−0.55	0.012	0.64
**Lower limb**
Peak power (W)	355 ± 38	419 ± 19	19.2 ± 12.3	<0.001	−2.21	338 ± 35	354 ± 33	4.8 ± 3.0	<0.001	−0.48	0.003	0.78
Peak power per body mass (W/kg)	5.6 ± 0.7	6.5 ± 0.4	16.6 ± 12.3	<0.001	−1.63	5.3 ± 0.5	5.4 ± 0.4	2.6 ± 3.4	0.007	−0.23	0.004	0.75
Maximal pedaling velocity (rpm)	170 ± 20	157 ± 16	6.8 ± 10.4	<0.001	0.74	166 ± 23	164 ± 21	−0.9 ± 9.4	0.584	0.10	0.285	0.27
Maximal braking force (N/kg)	7.9 ± 0.6	10.3 ± 0.8	30.8 ± 13.6	<0.001	−3.50	7.7 ± 0.6	8.7 ± 1	13.4 ± 13	<0.001	−1.25	<0.001	0.92

## Discussion

The main findings of the present study were that a 10-week elastic band training program improves COD, jumping, repeated sprint ability, and both upper and lower-body muscular strength and power in adolescent female handball players more compared to typical in-season training.

The present loads of the elastic band training program seem to elicit better force production through a greater range of motion and higher contraction velocities due to higher movement speeds and longer force application times (15, 30) as it increased almost all parameters. It had a large effect (ES≈1.7) upon maximal muscle strength in upper and lower body as shown by the increased 1-RM in bench press (47%) and half squats (22%), which was much more than the control group that enhanced with 10% and 6% (Table 4) resulting in a medium effect size between the two groups (ES = 0.59–0.67). This was in accordance with the review of Lopes, Machado (17) who demonstrated that elastic resistance training improves muscular strength. Several factors can explain this improvement, such as neural mechanisms and muscular innervation, such as adaptations in motor-unit activation, synchronization, and rate coding, rather than muscular hypertrophy, seem the most likely reasons for the performance improvement of the strength variables (5, 6, 14, 31). Also, these improvements in strength may be dependent on changes in body mass, which is not the case since we did not measure muscle volume. This can be considered among the limitations of this work.

The enhancement elicited by the elastic band training was even more visible in strength and power as indicated by the increases in peak power and maximal force in upper and lower body measured in the force-velocity tests. Furthermore, was the increased power found in the more handball related vertical jump performances in the elastic strength group compared to the controls (Table 4). No difference between the groups was found for the standing long jump, which may arguably be more dependent on coordinative aspects of performance than any pure strength or neuromuscular variables. Other forms of training, such as horizontal jump movements or plyometric training, may need to be considered to elicit horizontal jump improvements. Improvement in vertical jump performance is due to the nature of elastic band training in eliciting improvements in the rate of force production by allowing for force production through a greater range of motion. Like our result, Andersen, Fimland (6) found increases in both countermovement jump with and without arm swing after a 9-week strength training program using elastic bands in young female handball players. Conversely, using similar strength training with elastic band protocols in male elite handball, Aloui, Hermassi (5) found no significant change in vertical jump performance after 8-week elastic band training in junior male handball players. The discrepant findings from the present study could be explained by the intervention. First, the training period was shorter in (6), as they adopted a 9 -week training period with a rate of 3 sessions per week. As for (5), used a period of 8 weeks with two sessions per week compared to our research, which consists of 10 weeks (the longest period) between research and can be considered factors that contributed to. Second, the number of exercises of the lower limbs, as Andersen used one exercise for the lower limbs (Bulgarian squat), but our research used 4 exercises for the lower limbs (knee extension; knee flexion; half squat; and hip adduction), which explains the improvement in vertical jump performance.

Furthermore, the elastic band training enhanced COD performance, which is important to surpass the opponent in handball. The improved individual's force and power output are the mechanisms that enhanced the COD ability, which refers to a preplanned movement where no immediate reaction to a stimulus is required (32). Many studies revealed increases and decreases in COD performance after strength training with an elastic band (5, 6, 15). Indeed, Aloui, Hermassi (5) found increases in COD performance after 8-week strength training with elastic band in junior male handball players. Conversely, Andersen, Fimland (6) found no significant change in COD after 6-week elastic band training in young female handball players. The difference in findings are probably the result of the length of the intervention as 6 weeks of elastic band training seems not to induce enough to show significant differences, while eight or, as in present study ten weeks of elastic band training observed COD improvement (5.8%, Table 4).

This is the first study to demonstrate a positive large effect of elastic band strength training on repeated sprint ability performance (times) in adolescent female handball players. Controversy, fatigue index during the repeated sprint test remained unchanged. One possible explanation for the lack of signiﬁcant change in this parameter could be the poor reproducibility of this particular measure (33). To the authors' knowledge, no study has previously addressed the effects of elastic band training on repeated sprint ability performance in young female athletes. Using similar elastic band protocols in male elite handball players, Aloui, Hammami (16) found that 8 weeks of bi-weekly lower limb elastic band-based loaded plyometric training into the in-season regimen in junior male handball players improved best time and mean time in repeated COD scores after 8-week elastic band training. Repeated sprint ability is a complex quality related to both neuromuscular (i.e., acceleration and maximal sprint speed, e.g., neural drive or motor unit activation) and metabolic factors (23, 34). Thus, training strategies targeting the development of aerobic performance may account for an improvement in repeated sprint ability (35, 36). These improvements may be related to a boost in aerobic fitness and muscle buffer capacity that may promote faster PCr resynthesis (37, 38).

## Conclusion

In conclusion, the present study demonstrates that a 10-week in-season intervention based on elastic band training sessions twice a week can improve certain critical aspects of performance fitness in adolescent female handball players. This suggested that the improvement in COD, jump, power, strength and repeated sprint performance after a 10-week intervention was most likely due to the improvement in muscular power. Because of the relatively short duration of elastic band sessions, coaches and players can solicit higher physical demands than those presented in an official match-play (maximal aerobic power, changes of direction, and high-intensity actions), thus developing these components of handball fitness without greatly curtailing the regular training program where handball-specific technical and tactical skills are developed.

## Data Availability

The raw data supporting the conclusions of this article will be made available by the authors, without undue reservation.
